# An Effective Approach for Controller Placement in Software-Defined Internet-of-Things (SD-IoT)

**DOI:** 10.3390/s22082992

**Published:** 2022-04-13

**Authors:** Jehad Ali, Byeong-hee Roh

**Affiliations:** 1 Department of Computer Engineering, Ajou University, Suwon 16499, Korea; jehadali@ajou.ac.kr; 2 Department of AI Convergence Network, Ajou University, Suwon 16499, Korea

**Keywords:** SDN, controller placement problem, OpenFlow, k-means, ANP

## Abstract

The Software-Defined Networking (SDN) paradigm has transferred network intelligence from network devices to a centralized controller. Controllers are distributed in a network to eliminate a single point of failure (SPOF) and improve reliability and balance load. In Software-Defined Internet of Things (SD-IoT), sensors exchange data with a controller on a regular basis. If the controllers are not appropriately located in SD-IoT, the E2E latency between the switches, to which the sensors are connected, and the controller increases. However, examining the placement of controllers in relation to the whole network is not an efficient technique since applying the objective function to the entire network is a difficult operation. As a result, segmenting the network into clusters improves the efficiency with which switches are assigned to the controller. As a result, in this research, we offer an effective clustering strategy for controller placement in SDN that leverages the Analytical Network Process (ANP), a multi-criteria decision-making (MCDM) scheme. The simulation results demonstrated on real Internet topologies suggest that our proposed method outperforms the standard k-means approach in terms of E2E delay, controller-to-controller (C2C) delay, the fair allocation of switches in the network, and the communication overhead.

## 1. Introduction

By separating the data plane from the control plane, the SDN paradigm [1] transformed computer networks. SDN controllers may now instruct network devices using applications running on the controller, while the network itself is isolated from the applications [2,3]. With the SDN controller, the network does what the applications on it tell it to. Thus, network complexity is reduced since the logic is moved from the endpoints to the central software-defined network controller, which manages the underlying network from a single location. Due to these several advantages, an SDN-based architecture is used for the Internet of Things (IoT), which is known as SD-IoT [4,5,6,7].

When multiple controllers manage SD-IoT in an end-to-end (E2E) manner, then the frequent interchange of packets among the SDN controllers and switches affects the quality of service (QoS), i.e., E2E delay, if the switches are not properly assigned to the controllers. Hence, the switches’ allocations to the controller and placing the controller in SDN will influence the performance, which needs to be further explored; i.e., we need proper locations to place the controllers.

**Figure 1:**
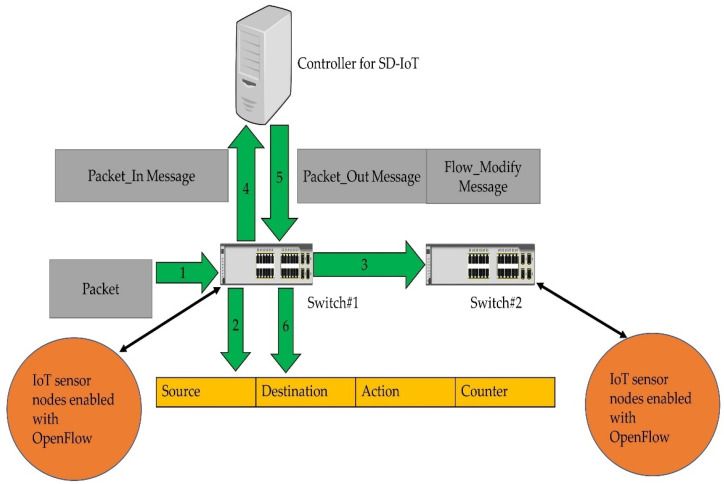
Packet processing in SD-IoT.

In SD-IoT, the SDN controller and the forwarding devices or switches are the two most important components. OpenFlow is used to demonstrate packet processing in SDN. The SDN controller connects all of the switches in the network. There is a new packet in the network, which is seen in Figure 1. SDN switches all store a flow table on their storage media. The access switch receives all packets sent by the IoT sensors as shown by step 1 in the figure. A packet will be forwarded on the switch’s output port if it matches an entry in a flow table which is denoted with steps 2 and 3. A table miss occurs if a flow entry is missing from the switch forwarding table. Step 4 indicates that a Packet-In message is issued to the controller whenever a table miss occurs. Switches transmit Packet-In messages to the controller, and they are subsequently followed by Packet-Out messages from the controller as shown with step 5 and 6. This is how Ethernet works. For example, using a Packet-Out message, a switch may be instructed to forward the packet or to apply instructions to it using a Flow-Modify message. Because of this, the switch’s operation is modified by the Flow-Modify message. As can be seen in Figure 1, messages are often exchanged between the switches and controllers. As a result, the message exchange and performance in SD-IoT are influenced by how the switches are assigned to the controller.

The SDN paradigm separates the control plane from the data plane, allowing for better network administration. If a single controller controls the whole E2E network, which is what the control plane logically depicts. There are various concerns, however, with the network having just one controller. For example, the network will go down if the controller fails (SPOF). As the number of switches assigned to a controller grows, the controller may become overburdened by the amount of flow requests generated by the IoT sensors in SDN. Similar to controller-to-switch delays increasing in size, so too will the network’s overall bandwidth requirements. Thus, an E2E network is made up of several controllers. Given an E2E network with a distributed set of controllers, such that each controller manages either one or more switches, the controllers maintain a consistent view of the global topology through the exchange of messages [8]. Therefore, a suitable partitioning of the network into multiple clusters is required in order to find suitable locations for the controllers [9]. However, the network partitioning brings a tradeoff among multiple metrics, such as reliability, latency, and load balancing [10]. Therefore, partitioning the network and finding a location for the controller in each cluster is another challenging issue [11]. The exploration of proper locations to be allocated to the controllers and the suitable assignment of the switches to these controllers in order to achieve an objective is known as the controller placement problem [9]. Hence, in this paper, we address the problem of controller placement in SD-IoT.

The remainder of the paper is organized as follows: In Section 2, we describe the related works. The introduction of the controller placement problem is given in Section 3. Our proposed scheme is illustrated in Section 4. The results and discussion are explained in Section 5. Section 6 concludes the paper and describes future research directions.

## 2. Related Works

Controller placement is a critical issue in SDN. Hence, several researchers have evaluated the problem of controller placement. Table 1 discusses the related works for controller placement in SDN and SD-IoT, in addition to the drawbacks of each approach, which pave the way for our proposal.

The placement of controllers in a topology depends on several criteria. However, propagation latency is one of the most important criteria considered by researchers in the literature. However, the definition of propagation latency made by researchers is not clearly defined. For example, in the most well-known k-means- and k-center- [12,13] based algorithms for the placement of controllers in SDN, propagation latency is defined based on the Euclidean distance (ED). However, the Euclidean distance is not an accurate representation of network topology, because the routers in a network are connected through physical links. In the recent literature [14], Haversine formulas [15,16] were adopted to calculate the distance between two nodes in a network topology. The propagation latency was then defined by dividing the distance over the signal propagation speed.

The placement of the controller in SDN is one of the most challenging problems and has attracted significant attention from both researchers and network engineers. The latency between switches and controllers is considered as the most important factor in controller placement [17,18,19]. High delays between switches and controllers can negatively affect the performance of controllers responding to network events within a reasonable amount of time. Studies in the literature [20,21] have considered propagation delays when assigning switches to SDN controllers.

These studies investigate how to lessen the propagation delay between switches and controllers using genetic algorithm and heuristic approaches. However, the genetic algorithm cannot guarantee the finding of the global maxima, and it needs a large population size, due to which the time of convergence is longer. Similarly, Heller et al. [13] proposed research on the placement of controllers in SDN corresponding propagation latency (where the average propagation and worst propagation delay are key considerations). The problem is devised as a facility location for which they adopted k-center. Likewise, Yao et al. [12] advanced the literature by considering the controller capacity in addition to the propagation latency. Hence, the placement was considered as an alternative to the capacitated problem with k-center [22].

The k-center- and k-means- [12,13] based approaches initialize the centers randomly, and, then, in each iteration, they assign the switches to new centers until there is no change in the clusters. However, this does not guarantee the minimum propagation delay. For example, with a new center, the delay is found to be higher than in the previous cluster as mentioned in [14].

Similarly, controller placement in IoT based on SDN is explored in [23], leveraging a sub-modularity approach based on a heuristic approach. However, the authors did not investigate E2E latency. Moreover, the results are not demonstrated in real Internet topologies.

### Contributions and Research Gap

Previous works used k-means or k-center [12,13] to find the center of the network topology, and then the placement was selected based on the center point. However, k-means does not provide effective results, and it is not effective because, sometimes, more iteration results do not minimize the E2E delay. Other works do not evaluate several metrics while computing the E2E delay using the real emulated environment from Mininet as discussed in Table 1. Moreover, the IoT-based controller placement approach [23] is not evaluated regarding the E2E delay in network topologies in relation to several parameters that contribute to the E2E delay. The contributions of our proposed approach are as follows:

1. We formulate the problem of controller placement in SD-IoT with ANP MCDM and provide a system model for it.
2. Then, we demonstrate it with a mathematical explanation in OS3E topology.
3. Our demonstration is based on multiple metrics, which contribute to the E2E delay.
4. The demonstration results are tabulated for explanation.
5. In addition, we also use Mininet to compute the factors that result in the E2E delay.
6. Moreover, we perform simulations on several network topologies and compare the standard k-means algorithm for controller placement.
7. We compare the results in terms of the E2E delay between the controllers and the switches, among the controllers, the communication overhead, and the fairness index, with k-means algorithms regarded as benchmarks for the controller placement problem.

**Table 1:** Literature works and limitations.

Related Works	Limitations
[12,13]	These works do not offer the minimum E2E delay. The reason being that both are primarily based on random placements. Moreover, ED is utilized for propagation delay, which represents real topologies.
[14]	In [14], the authors consider Matlab simulations. However, the results of Mininet are not considered. Moreover, the communication overhead and the results of the controller-to-controller latency are not evaluated.
[17]	An enhanced version of k-means. However, multiple network parameters such as queuing delay are not considered when computing the E2E delay.
[18]	The propagation latency is considered by calculating the geographical distance. However, other metrics are not considered, such as queuing latency, path computation, and link utilization.
[19]	Various factors contributing to the E2E delay are not discussed. The evaluations do not reflect a real network infrastructure.
[21]	Heuristic method of controller placement in SDN. However, queuing and path computing latency are not considered. Moreover, results from Mininet environment are not evaluated.
[22]	A location allocation problem, also known as the NP-hard, but it is not evaluated in the context of SDN.
[23]	A controller placement approach for SD-IoT. However, the results are not evaluated in real Internet topologies. Moreover, there is no comparison of the delay between the switches in the network and the controllers.

## 3. Controller Placement Problem Effect on the QoS in SD-IoT

**Figure 2:**
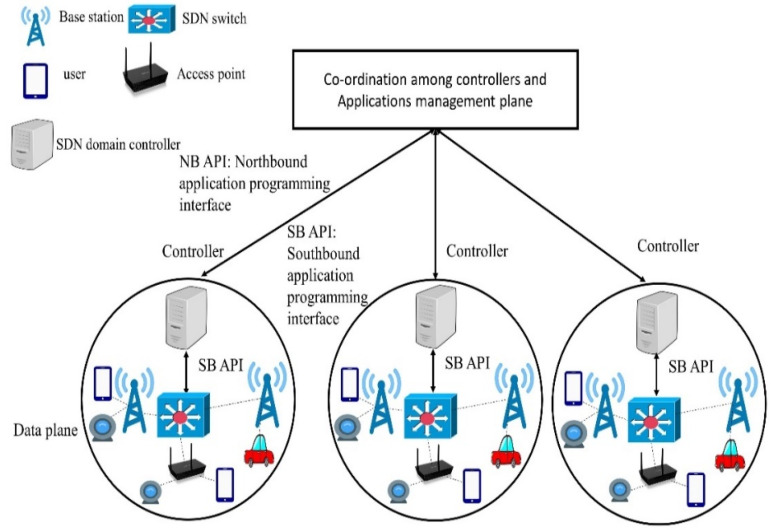
SD-IoT architecture.

In SD-IoT, a single controller cannot manage an E2E network because (1) a single controller leads to a single point of failure and (2) if the number of switches attached to a controller increase, the number of messages can overwhelm the performance [24] of the controller; i.e., a single controller hinders scalability in SDN. Due to these reasons, multiple controllers are placed in SDN. However, when there are multiple controllers to be placed, then the proper number of switches should be assigned to them because when a packet comes on the ingress port of a switch, the flow table is searched for a matching flow entry according to the incoming packet header information. In case there is no flow entry, then the packet is forwarded to the SDN controller. The SDN controller then finds the destination and inserts a flow entry in the switch. Therefore, if the switches are not properly assigned to the controller, then this process increases the delay. Figure 2 shows an example of SD-IoT architecture. The SDN switches communicate through OpenFlow with the domain controllers distributed in the geographical area. As there is frequent communication between the switches and the controller due to newly arrived packets from the IoT sensors attached to the SDN switches, the proper allocation of the switches to each controller results in a reduction in the E2E delay.

**Figure 3:**
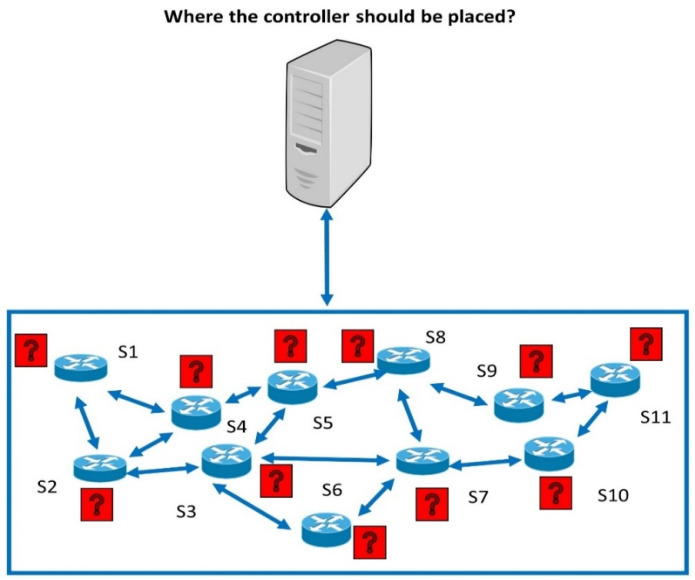
The controller placement effect on the QoS.

Figure 3 shows SDN with data plane switches S1, S2, S3, …, S11 distributed in the network; the controller placement should be selected to reduce the delay between the switches and the SDN controllers. The exploration of proper locations to be allocated to the controllers and the suitable assignment of the switches to these controllers to achieve an objective is known as the controller placement problem (CPP) [25]. The objectives are to reduce the E2E delay between the switches and the controller and to fairly distribute the switches in the network. Both objectives result in a reduction in the E2E delay. Hence, we address the allocation of the switches to the controller in this paper.

## 4. Proposed Approach for Controller Placement

In this section, we describe the procedure carried out to place the controllers in the clusters of the SDN topology. The SDN network is represented with *G = (V*, *E)*, and the clusters are denoted with k_1_, k_2_, k_3_, …, kn. The switches in each cluster are identified with *S*_1_, *S*_2_, *S*_3_, …, *S*_M_. The criteria set based on which the controller placement is selected is denoted with *C*_1_, *C*_2_, *C*_3_, …, *C*_N_.

**Figure 4:**
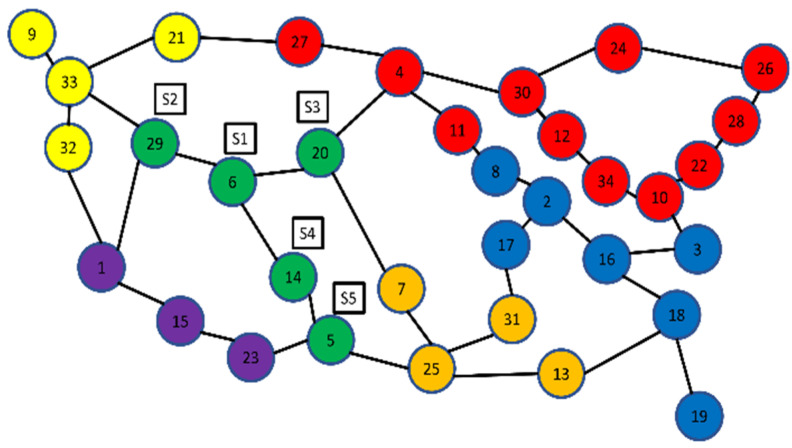
Placement of controllers in OS3E demonstration with ANP model.

The ANP MCDM problem is modeled by first setting our goal or objective, then defining the parameters for criteria or sub-criteria for evaluation, and finally ranking the alternatives. Figure 4 shows an OS3E topology and the switches in each cluster; e.g., in the green cluster, we can observe that there are five switches. In this study, our objective is to select the optimum placement for a controller in a cluster. For example, in green cluster i.e., S1, S2, S3, S4 and S5, the place will be selected through ANP. Similarly, other numbers with nodes such as 9, 33, 21, 32 denotes another cluster, where these switches will be ranked through a criteria and son on. The criteria parameters are hop count (HC), propagation latency (PL), queuing latency [14], path computation latency, and link utilization (LU) [26] as shown in Table 2. Switches are the alternatives to be ranked, available in a cluster of SDN topology, and the controller will be piggybacked upon the switch computed using the ANP model. Equations (1) and (2) denote the criteria and the alternatives.

*C* = (*C*_1_, *C*_2_, *C*_3_, …, *C*_N_) (1)

*S* = (*S*_1_, *S*_2_, *S*_3_, …, *S*_M_) (2)

### 4.1. Pairwise Comparison Matrices Calculations

Each switch is compared with respect to the criteria, i.e., PD and HC, queuing delay, and LU. For example, for *C*_1_ criterion, which is PD, a comparison is made with respect to all switches. Then, the same is carried out for switch *C*_2_*,* and so on. An example of the comparison matrix is shown below. We also refer to it as the generic comparison matrix denoted with Equation (3). Herein, the value of 1 shows that two switches are equally likely to be important with respect to a specific criterion parameter. The nonreciprocal and reciprocal values indicate the relative importance of the row and column components, respectively. For example, in Equation (4), $a_{\left(1,\;5\right)}=\frac{1}{6}$ shows that *S*_5_ is significantly to remarkably more important than *S*_1_. The relative importance values are important on a 9-point scale, where the values of comparison range from 1 to 9, with 9 showing high importance of one criterion over another, and 1 showing low significance of one criterion over another.

Then, Equation (4) is normalized according to Equation (5). Equation (5) shows that every column in matrix Equation (4) is summed up, and each value is divided by the sum of the total values of the column according to matrix Equation (5).

The eigenvector *X* is obtained for normalized matrix Equation (5) by carrying out the steps illustrated in Equation (6).

(3) $$A_{k=1}=\left[\begin{array}{ccccc} 1 & a_{\left(1,2\right)} & a_{\left(1,3\right)} & \cdots & a_{\left(1,n\right)}\\ \frac{1}{a_{\left(1,2\right)}} & 1 & a_{\left(2,3\right)} & \cdots & a_{\left(2,n\right)}\\ \frac{1}{a_{\left(1,3\right)}} & \frac{1}{a_{\left(2,3\right)}} & 1 & \cdots & a_{\left(3,n\right)}\\ \vdots & \vdots & \vdots & \ddots & \vdots \\ \frac{1}{a_{\left(1,n\right)}} & \frac{1}{a_{\left(2,n\right)}} & \frac{1}{a_{\left(3,n\right)}} & \cdots & 1 \end{array}\right]$$

(4) $$\left[\begin{array}{cccccc} & S_{1} & S_{2} & S_{3} & S_{4} & S_{5}\\ S_{1} & 1 & \frac{1}{3} & \frac{1}{3} & 3 & \frac{1}{6}\\ S_{2} & 3 & 1 & 1 & 6 & \frac{1}{3}\\ S_{3} & 3 & 1 & 1 & 6 & \frac{1}{3}\\ S_{4} & \frac{1}{3} & \frac{1}{6} & \frac{1}{6} & 1 & \frac{1}{9}\\ S_{5} & 6 & 3 & 3 & 9 & 1 \end{array}\right]$$

(5) $${\left[\begin{array}{ccc} \frac{a_{\left(1,1\right)}}{\sum_{i=1}^{n}a_{\left(i,1\right)}} & \cdots & \frac{a_{\left(1,n\right)}}{\sum_{i=1}^{n}a_{\left(i,n\right)}}\\ \vdots & \ddots & \vdots \\ \frac{a_{\left(n,1\right)}}{\sum_{i=1}^{n}a_{\left(i,1\right)}} & \cdots & \frac{a_{\left(n,n\right)}}{\sum_{i=1}^{n}a_{\left(i,n\right)}} \end{array}\right]}_{\;}$$

(6) $$X_{i}=\frac{1}{n}\sum_{j=1}^{n}a_{\left(i,j\right)},\;\mathrm{where}\;i=1,\;2,\;3,\;\ldots ,\;n$$

### 4.2. Finding the Consistency Index

To determine whether the judgments made while making the pairwise matrix are coherent, the *CI* and *CR* values must be obtained. However, before assessing consistency, the consistency measure *(CM)* vector needs to be determined. The *CM* vector is a precondition for the computation of *CI* and *CR*. The consistency rate is calculated corresponding to Equation (8). *M_j_* indicates the row values in the comparison matrices, i.e., Equations (3) and (4). Moreover, *X* and $\;x_{i}$ signify the eigenvector and the resultant element of the eigenvector, respectively, as revealed in Equation (6). *M_j_* and *X* are multiplied and then divided by the component in the eigenvector corresponding to *M_j_*. The process carried out to locate the *CM* [27] is demonstrated in Equation (7). The average of the *CM* vector is taken to compute $\lambda_{max}$, which is denoted in Equation (9).

(7) $$\left[\begin{array}{c} Y_{1}\\ Y_{2}\\ Y_{3}\\ ↓\\ Y_{n} \end{array}\right]=\left[\begin{array}{ccccc} a_{11} & a_{12} & a_{13} & \to & a_{1n}\\ a_{21} & a_{22} & a_{23} & \to & a_{2n}\\ a_{31} & a_{32} & a_{33} & \to & a_{3n}\\ ↓ & ↓ & ↓ & ↓ & ↓\\ a_{n1} & a_{n2} & a_{n3} & \to & \;a_{nn}\; \end{array}\right]\times \left[\begin{array}{c} x_{1}\\ x_{2}\\ x_{3}\\ ↓\\ x_{n} \end{array}\right]$$

(8) $$Y_{j}=\frac{M_{j}\times X}{x_{i}},\;\mathrm{where}\;j=1,\;2,\;3,\;\ldots ,\;n$$

(9) $$\lambda_{max}=\frac{1}{n}\overset{n}{\underset{j=1}{\sum }}Y_{j}$$

Consistency Index: The inconsistency [27] of the pairwise comparison matrix for an element, i.e., a switch, in the cluster is represented with *CI*. Hence, the *CI* of the comparison matrix for *C*_1_, i.e., propagation latency, is calculated using Equation (10) by inserting the value of $\lambda_{max}$. The $\lambda_{max}$ value is inserted into Equation (10).

(10) $$CI=\frac{\left(\lambda_{max}-n\right)}{\left(n-1\right)}$$

In Equation (10), *n* is the comparison matrix’s criterion number for controller selection. The dependability of the pairwise comparison matrix is validated by computing the consistency ratio (*CR*). Equation (1) is used to compute the *CR* as shown in Equation (11). The index ratio is denoted by the ratio index (*RI*) in Equation (11). Table 3 yields the value of *RI*, which is dependent on the matrix’s order. Hence, if the matrix’s rank is three (the actual number of switches being compared), a value for *RI* equal to three is inserted from Table 3. The number of factors considered in this situation is five switches. As a result, a value equal to 5 is added from Table 3. The *CR* is calculated by inserting the *CI* value from Equation (10) into Equation (11).

(11) $$CR=\frac{CI}{RI}$$

For inconsistent judgements of the comparison matrix, a *CR* of 0.1 or less is acceptable (*CR* ≤ 0.1); otherwise, the inconsistency is regarded as strong, and paired assessments must be performed again to fulfill the criteria.

### 4.3. Calculation of the Final Placements (Alternatives)

As demonstrated in Equation (6), the eigenvectors (which reveal the weight of each criterion in relation to each alternative and vice versa) are computed and displayed in an unweighted super-matrix. The unweighted super-matrix is then modified to become col-um stochastic, with each column’s sum equal to one. The matrix becomes a weighted super-matrix as a result of this process. The unweighted super-matrix is identical to the weighted super-matrix; however, the weighted super-matrix is column stochastic, but the unweighted super-matrix is not. *S*_1_, *S*_2_, *S*_3_, *S*_4_ and *S*_5_ indicate the priority values of the switches where a controller should be positioned. *X*_1_*, X*_2_*, X*_3_, *X*_4_ and *X*_5_ are the eigenvectors corresponding to *S*_1_, *S*_2_, *S*_3_, *S*_4_ and *S*_5_, respectively, to reflect the priority values of the switches where a controller should be placed. The computation of the limit super-matrix is the next step in the ANP model to acquire the final stable weights of the alternatives.

The weighted super-matrix is processed by scaling it up to a higher power until it converges to a stable matrix. A limit super-matrix is a stable matrix. The weights of the options and the criteria, i.e., the final prioritized values, are shown in the limit matrix. The final weights measured against each element in the criterion and alternative clusters are included in the limit matrix, which is the outcome matrix. This is calculated using a weighted super-matrix with values increased to the power of 2*k* in order to obtain the same value for each row, where k may be any random integer [28,29,30]. The pairwise comparisons of all matrices are summarized by the limit super-matrix. This also depicts the components’ indirect link. The result of the limit super-matrix is shown in Table 4, Table 5, Table 6, Table 7, Table 8 and Table 9, with the greater values representing the standing alternatives. The S. No in each table denotes the number of the eigenvector corresponding to the switch *S*. Table 4 shows that *S*_4_ has the largest weights, making it the best switch to piggyback a controller. *S*_1_, *S*_5_, *S*_2_ and *S*_3_ are the next best controllers based on their final weights determined from the limit super-matrix. According to the findings, *S*_4_ has a high weight value; hence, the SDN controller should be installed on that switch. The final alternative weights for the second cluster, the yellow cluster, are shown in Table 5. Similarly, Table 6 displays the weights for controller placement in the purple third cluster. The findings for cluster 4 are shown in Table 7. Table 8 and Table 9 show the weights of the switches appropriate for controller placement in the red and blue clusters, from high to low (clusters 5 and 6).

## 5. Simulation Results and Discussion

We evaluated the assignment of switches to controllers in four topologies, i.e., OS3E, US_Net, Abilene, and Interoute topologies, utilizing Mininet [31] and Matlab. The details of these topologies are shown in Table 10. Table 10 shows the number of nodes and edges in each topology. The simulations were conducted in the following steps: (1) We obtained the topologies from the topology zoo dataset. (2) In the second step, we calculated the propagation latency, queuing latency, PC latency, hop count, and link utilization. (3) Then, k-means was applied to initially form clusters in each topology. (4) Finally, the controller placement was selected with respect to the ranking of the switches in each cluster leveraging ANP.

**Figure 5:**
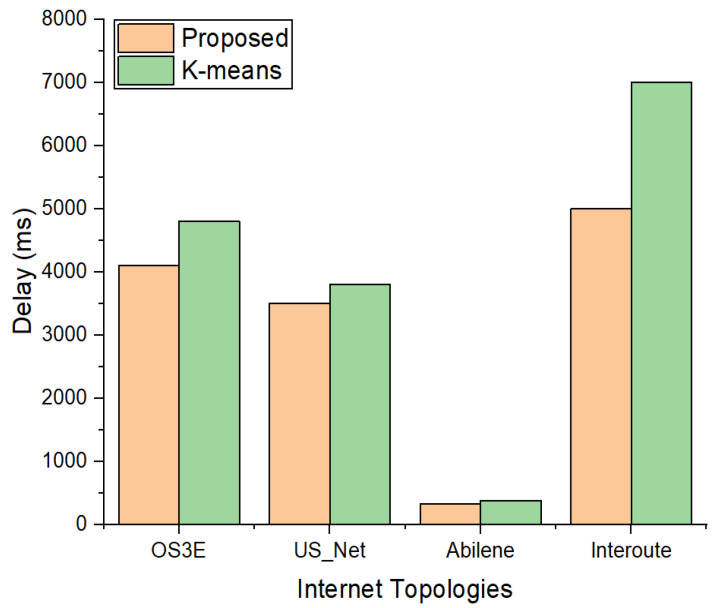
Comparison of E2E delay between proposed scheme and k-means with 6 clusters.

Figure 5 shows the E2E delay (total) calculated for the four topologies, i.e., Abilene, OS3E, Interoute, and US_Net, which were divided into six partitions. We calculated the total E2E delay using Equation (12), which is a combination of PD, QD and PC, i.e., propagation delay, queuing delay, and path computation delay, respectively. The E2E delay results were compared with the k-means clustering algorithm. The graph shows that ANP based clustering has a smaller E2E delay in the four topologies than that of k-means. The reason for this is that k-means selects the initial placements randomly. However, the ANP selects the most appropriate placement for the controller, which gives less E2E delay with respect to all switches in each partition/cluster. Furthermore, the results of the four topologies reveal that the percentage of E2E delay for Abilene is smaller than those for US_Net, Interoute, and OS3E, because, in this topology, the propagation latency is lower than the others, which contributes to the E2E delay.

Delay_total_ = PD + QD + PC (12)

**Figure 6:**
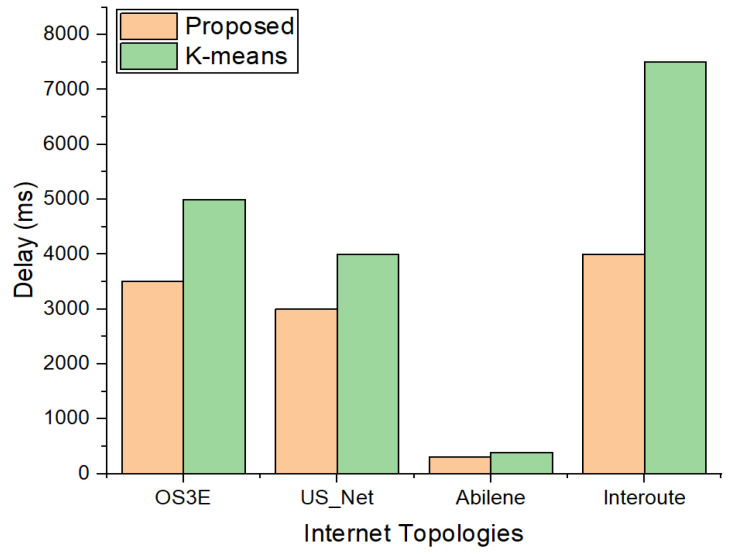
Comparison of E2E delay between proposed scheme and k-means with 7 clusters.

Figure 6 shows the results of the four topologies with seven clusters. It is evident that k-means with seven partitions results in a slight increase in the delay, because with more repetitions, k-means does not guarantee that new clusters will result in a reduced E2E delay. However, the proposed approach evaluates all the alternatives to reduce the E2E delay. Hence, we can see that, even with an increasing number of nodes and links in the topologies, the E2E delay is decreased in our proposed scheme.

**Figure 7:**
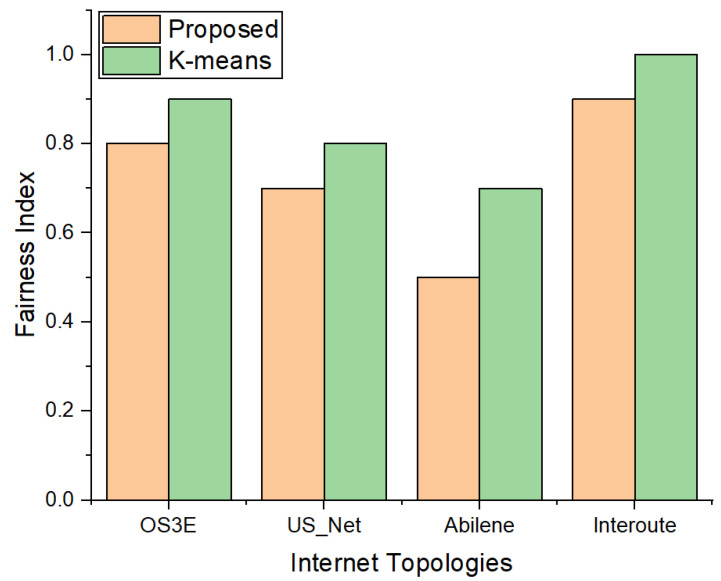
Comparison of the fairness index between k-means and proposed approach.

We also calculated the fair distribution of the switches using our proposed approach and k-means; i.e., the number of maximum switches in a cluster was divided by the number of clusters formed through k-means and our proposed scheme. A lower value indicates that there is a smaller number of switches allocated to each cluster with respect to the number of controllers for the topology. Figure 7 shows the results of the fairness index. We can see that the proposed scheme fairly allocates the switches in each cluster in the four topologies, even with the increasing number of nodes and links.

**Figure 8:**
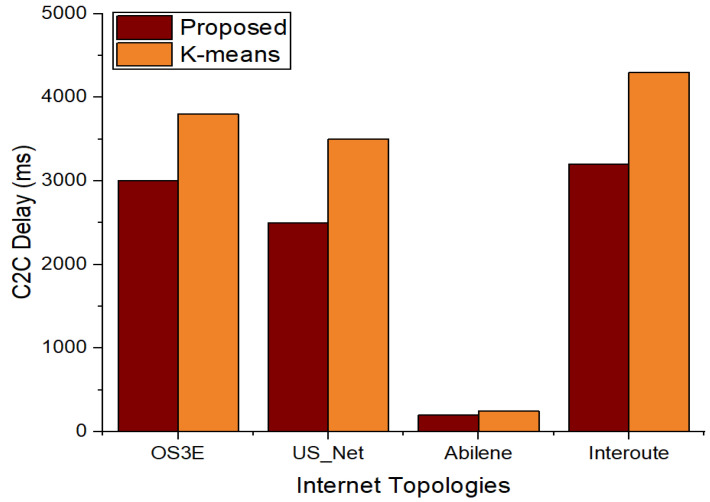
Comparison of C2C delay between proposed scheme and k-means with 6 clusters.

**Figure 9:**
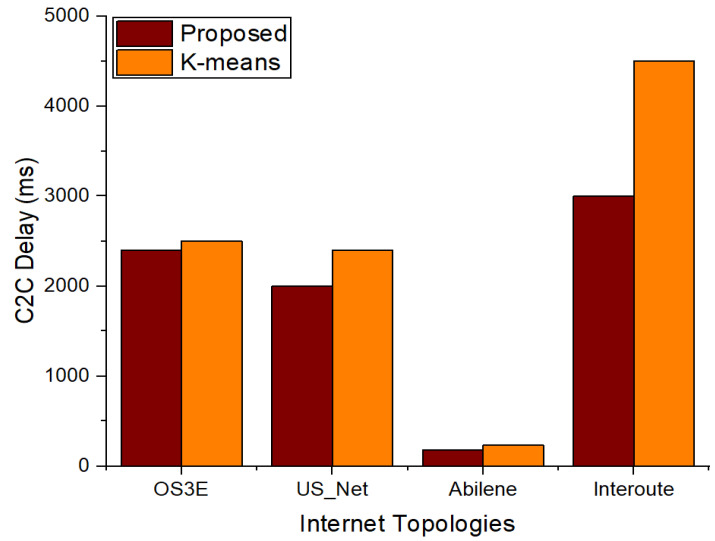
Comparison of C2C delay between proposed scheme and k-means with 7 clusters.

The findings of the controller-to-controller (C2C) delay with our suggested method and the benchmark model in the four topologies with six and seven clusters are shown in Figure 8 and Figure 9, respectively. Figure 8 illustrates that the suggested approach has a shorter C2C delay in all topologies. This is due to the right positioning of the controllers in relation to the five criteria that were used to rank the controllers and contribute to the delay reduction. The findings of the C2C delay with seven clusters are shown in Figure 9. Figure 9 shows that the C2C latency of the Interoute and OS3E topologies rises rather than decreases for k-means. This is due to the initial random placement of the controllers using k-means. However, as compared to the benchmark k-means algorithm, with an increase in clusters, the suggested technique has a lower C2C delay.

**Figure 10:**
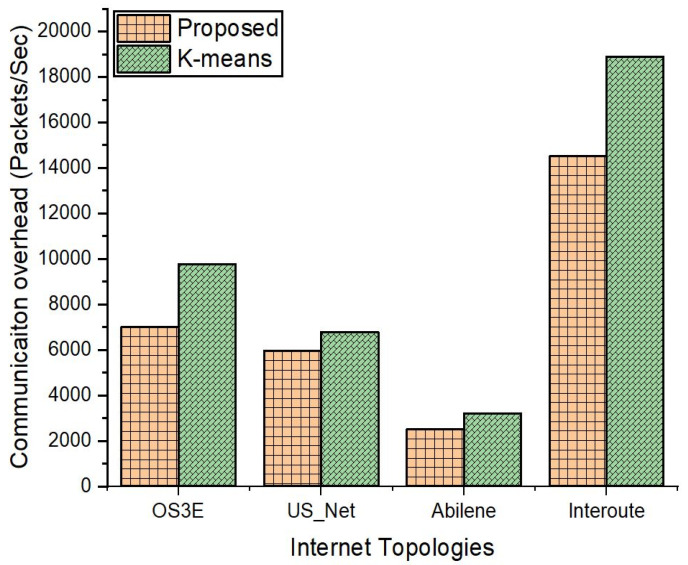
Communication overhead between the switches and controllers.

**Figure 11:**
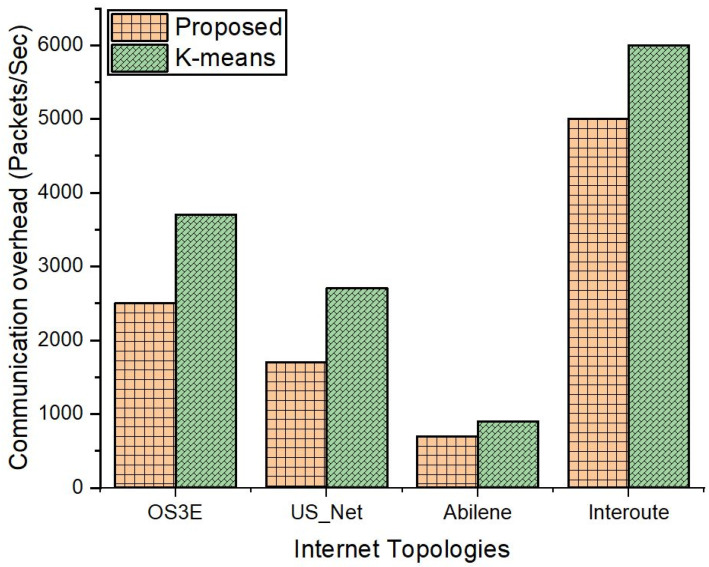
Communication overhead among controllers (CT-CT).

Figure 10 and Figure 11 show the comparison results of the communication overhead between our proposed scheme and k-means. The communication overhead is represented as the number of packets transferred between the switches and the controller (SW-CT), which is shown in Figure 10, and controller to controller (CT-CT), which is shown in Figure 11. We evaluated it in the four topologies for the proposed scheme and compare it with the benchmark models. Figure 10 reveals that the communication overhead is high for k-means as compared to our proposed method. As more packets are exchanged, there will be more frequent interactions with the controller; hence, there will be more computations performed by the controller, which also results in a higher delay as shown in the previous results.

Figure 11 reveals that the packets exchange, which is a measure of the communication overhead in our experiment for CT-CT. The results of the four topologies indicate that the proposed scheme surpasses k-means in terms of communication overhead among the controllers. The results of the Interoute topology show that the CT-CT communication overhead is high compared to the other topologies, namely, Abilene, OS3E, and US_Net. As the SD-IoT network size grows, more controllers will be placed in the network, hence resulting in a larger communication overhead.

## 6. Conclusions

The SDN paradigm shifts control to a centralized omniscient controller. The controller, however, creates a bottleneck due to the enormous amount of message exchanges between the switches and the SDN controller in SD-IoT. As a result, inappropriate switch assignment to the controller reduces performance. Previously, the k-means method was often employed in SDN controller placement selection. However, k-means has the drawback that, even after numerous rounds of applying the method, the E2E latency is not reduced. As a result, in this research, we employed ANP in conjunction with the k-means method for controller placement. Finally, we tested the suggested approach on real-world Internet topologies and compared the results to those of a typical k-means algorithm. For controller placement, our suggested method outperforms the k-means algorithm in terms of the E2E delay between the switches and the controllers in the clusters, as well as among the controllers distributed in the topology, the fairness index, and the communication overhead.

Future studies will include an evaluation of the suggested technique for a variety of parameters, such as energy efficiency and load balance. Furthermore, the suggested technique is generic, allowing it to be evaluated in a range of situations for controller placement in 5G and beyond networks. As a result, more research will be conducted to address these issues.

## Figures and Tables

**Table 2:** Alternatives and criteria.

Criteria	Criterion Symbols	Alternatives
Propagation latency	*C*_1_	*S*_1_
Hop count	*C*_2_	*S*_2_
Queuing latency	*C*_3_	*S*_3_
PC latency	*C*_4_	*S*_4_
Link utilization	*C*_5_	*S*_M_

**Table 3:** RI values for number of criteria.

Criteria	1	2	3	4	5	6	7	8	9	10
Ratio index	0.00	0.00	0.58	0.90	1.12	1.24	1.32	1.41	1.45	1.49

**Table 4:** Weights of the switches for the controller placement in cluster 1 (green).

S. No.	Criterion Symbols	Limit Super-Matrix Weight
*X*_1_	*S*_1_	0.18
*X*_2_	*S*_2_	0.04
*X*_3_	*S*_3_	0.03
*X*_4_	*S*_4_	0.20
*X*_5_	*S*_5_	0.05

**Table 5:** Weights of the switches for the controller placement in cluster 2 (yellow).

S. No.	Criterion Symbols	Limit Super-Matrix Weight
*X*_9_	*S*_9_	0.19
*X*_33_	*S*_33_	0.30
*X*_21_	*S*_21_	0.18
*X*_32_	*S*_32_	0.17

**Table 6:** Weights of the switches for the controller placement in cluster 3 (purple).

S. No.	Criterion Symbols	Limit Super-Matrix Weight
*X*_15_	*S*_15_	0.40
*X*_1_	*S*_1_	0.04
*X*_23_	*S*_23_	0.04

**Table 7:** Weights of the switches for the controller placement in cluster 4 (dark yellow).

S. No.	Criterion Symbols	Limit Super-Matrix Weight
*X*_7_	*S*_7_	0.12
*X*_31_	*S*_31_	0.14
*X*_25_	*S*_25_	0.50
*X*_13_	*S*_13_	0.13

**Table 8:** Weights of the switches for the controller placement in cluster 5 (red).

S. No.	Criterion Symbols	Limit Super-Matrix Weight
*X*_27_	*S*_27_	0.18
*X*_4_	*S*_4_	0.16
*X*_11_	*S*_11_	0.40
*X*_30_	*S*_30_	0.45
*X*_12_	*S*_12_	0.48
*X*_34_	*S*_34_	0.52
*X*_10_	*S*_10_	0.60
*X*_22_	*S*_22_	0.41
*X*_28_	*S*_28_	0.50
*X*_26_	*S*_26_	0.55
*X*_24_	*S*_24_	0.56

**Table 9:** Weights of the switches for the controller placement in cluster 6 (blue).

S. No.	Criterion Symbols	Limit Super-Matrix Weight
*X*_8_	*S*_8_	0.03
*X*_2_	*S*_2_	0.08
*X*_17_	*S*_17_	0.09
*X*_16_	*S*_16_	0.20
*X*_3_	*S*_3_	0.10
*X*_18_	*S*_18_	0.07
*X*_19_	*S*_19_	0.02

**Table 10:** Network topologies.

No	Name	Nodes	Edges
1	Abilene	11	14
2	US_Net	24	42
3	OS3E	34	41
4	Interoute	110	149

## Data Availability

Not applicable.
